# A Case of Idiopathic Primary Abscess of the Orbit

**Published:** 1874-11

**Authors:** Swan M. Burnett

**Affiliations:** Knoxville, Tennessee


### A CASE OF IDIOPATHIC PRIMARY ABCESS OF THE ORBIT.

BY SWAN M. BURNETT, M.D., OF KNOXVILLE, TENNESSEE.

A negro boy, twelve years of age, was sent to me on the first of September, 1874, for examination, on account of an œdema of the lower lid of the left eye. He was in perfect health, and the eye gave him no pain. On examination, I found nothing noticeable except the œdema and some tenderness at and about the inner canthus. I ordered the parts to be painted with tinct. iodine, and told him to return next day.

On his return, not only had the swelling of the lower lid not abated, but there was also œdema of the upper lid, and some increase of tenderness at the inner canthus. Eyeball freely movable, but little redness and injection of the conjunctiva. Ordered the painting with iodine to be extended to the upper lid.

I did not see him again for two days. At that time the swelling had almost entirely subsided, and there had been some discharge of matter, he said. Indeed, I found some pus lying on the inner canthus and a small opening in the caruncle.

Examining the parts now, I found a tumor about the size of a small filbert in the orbit at the upper and inner corner. It was elongated, running back some distance into the orbit, but I could, by pushing my finger back, easily feel it in its entirety. By making pressure upon this tumor, a quantity of pus issued from the opening I had seen in the caruncle. Pressure on it gave him some, but not excessive, pain.

I did not see him again for several days. The tumor had now diminished to a very small size, and the discharge had stopped.

I met him on the street a day or two since entirely well. He had met with no injury to the eye, and there had been no inflammation, erysipelatous or otherwise of any of the neighboring tissues, the two most prolific causes, according to the text books, of abcess in the orbital cavity.

The interest attached to the case consists in this fact, and in the very mild and harmless course it ran; for orbital abcesses, as a rule, are very dangerous to the integrity of the eye.

In such a case as this, there might be some difficulty in making a differential diagnosis. It might be mistaken for an abcess of the lachryneal sac, but when it is remembered that the swelling in lachrymal abcess is external to the orbit, and below rather than above the canthus, the difficulty vanishes at once.

